# Malaria in Animals

**Published:** 1896-03

**Authors:** 


Malaria in Animals (from the same article). Malaria cannot be designated as an epizooty, since its presence depends to a very high degree on the neighborhood, and we are not at all certain whether the germs can be transported to neighborhoods which were originally free from it. I hold that the occurrence of genuine malaria in animals is still wholly unproven, but the English veterinary surgeons in Cape Colony designate a number of undoubtedly contagious diseases as malaria, and I must here at least mention the names of these epidemics. To enter upon a minute description of these diseases is impossible, particularly as I am acquainted with none of them from personal observation. I shall here merely call attention to those points in the information accessible to me which stand in contradiction to what is known about the origin of human malaria, in the hope of directing the attention of readers more conversant with the subject to the points which are still awaiting a thorough examination.

In the first place, I mention biliary fever or malarial jaundice in the horse. According to Hutcheon (Report of the Department), it is enzoötic in certain districts and takes such a violent course that it only responds to treatment when taken within the first twenty-four hours. It is most prevalent on the east coast, from midsummer (January, February) until the beginning of winter (May). This last characteristic corresponds exactly to the observations in the case of human malaria. The fact, also, that the disease is most prevalent in the eastern districts along the coast agrees with the well-established observation that malaria in the human family is confined in South Africa almost entirely to the same district. On the other hand, the statement that a cure by medical treatment is only possible within the first twenty-four hours does not at all agree with experience in regard to the course of malaria in the human family.

As a second disease, I would mention redwater in cattle. This disease takes its name from the bloody condition of the urine which is always present during sickness. The liver and gall are said to show very marked symptoms of disease also. This epidemic occurs chiefly in the rainy season, when the cattle are driven from the highlands down into the plains. It never occurs when the cattle are removed from lowlands to the highlands. The disease is said to be spreading further and further, and, in spite of all the regulations which have been placed upon the cattle trade, it is finding its way among cattle from infected districts into the grass lands of Cape Colony. Many hundred thousand pounds are lost annually by the ravages of this disease. According to Hellier, animals which have been raised on farms infected with the disease seem to have acquired immunity against it by some process of natural inoculation, possibly in some cases before they were born, for cattle raised in the neighborhood remain perfectly free from it, while imported animals are pretty sure to fall sick and die in the course of a month. This single observation seems to argue pretty positively against identifying redwater with any form of malaria or bringing it in any way in connection with the same. In the case of man, malaria always produces an increased tendency to fall a prey to some new disease, particularly when the patient has not been long afflicted. Again, people who have grown up in districts subject to fever are just as liable to fall sick with malaria as new-comers. Only in the case of the former the milder and chronic forms of the disease are more frequent than the pernicious and the acute. Here, however, we find the statement that animals which have become full grown in the infected districts enjoy full immunity against the epidemic. This fact can be no more easily accounted for on the basis of a genuinely contagious disease than on the basis of malaria, and that redwater is a real infectious disease seems to follow from the circumstance that, in spite of all the restrictions put upon the cattle trade, the disease is spreading further and further. In the case of malaria this fact cannot be accounted for.

Hellier reports that the Boers give "dips" (probably molasses made from grape juice) internally as a means of prevention. Hutcheon is very skeptical as to the possibility of finding a real preventive, but hopes that a better method of treatment may be found. Borthwick claims to have obtained results with certain medicines, while Hellier knows no method of treatment with specifics upon which he can depend. There is evidently great disagreement and unclearness in the method to be pursued, and we are justified in drawing the conclusion that medicine and natural immunity have been effective only in isolated cases.

Borthwick reports another observation which can be interpreted in the sense that redwater has nothing to do with malaria. He tried to diagnose previous stages of the disease in an infected herd, and found that the animals had a high temperature several days before an ordinary observer noticed it.

He treated the herd with his remedy:

| | |
|---|---|
| ℞.—Raw lin. ol. . . . . . . . | 1 quart bottle. |
| Acid carbol. pur. . . . . . | 60 drops. |
| Natr. subsulphuros. . . . . | 1 ounce. |
| Cal. chloric. . . . . . . . | 1 ounce. |

Given as a single dose, repeated the second day, which, as one can see, contains elements destructive to bacteria to a high degree, and lost out of thirty-two only two animals.

I have also been unable to make microscopical investigations in all these diseases which are regarded as forms of malaria by English veterinary surgeons. And yet no method of investigation is better adapted than this to determine the question whether we have to deal with a real animal malaria or not. The characteristic plasmodium of the blood-cells would then certainly be found. A certain degree of skill would be necessary for this investigation, because the important changes in the red and white blood-corpuscles in many infectious diseases might easily deceive an inexperienced man in regard to plasmodium.

